# Kinetics and Mechanisms of Phosphorus Adsorption in Soils from Diverse Ecological Zones in the Source Area of a Drinking-Water Reservoir

**DOI:** 10.3390/ijerph121114312

**Published:** 2015-11-10

**Authors:** Liang Zhang, Hugo A. Loáiciga, Meng Xu, Chao Du, Yun Du

**Affiliations:** 1 Institute of Geodesy and Geophysics, Chinese Academy of Sciences, Wuhan, Hubei 430077, China; E-Mails: dream0131@asch.whigg.ac.cn (M.X.); cdu@whigg.ac.cn (C.D.); duyun@whigg.ac.cn (Y.D.); 2 Department of Geography, University of California, Santa Barbara, CA 93106, USA; E-Mail: hugo.loaiciga@ucsb.edu

**Keywords:** phosphate, Danjiangkou reservoir, south-to-north water transfer projects (SNWTP), rate-controlling step, pseudo-second-order model, non-linear regression analysis

## Abstract

On-site soils are increasingly used in the treatment and restoration of ecosystems to harmonize with the local landscape and minimize costs. Eight natural soils from diverse ecological zones in the source area of a drinking-water reservoir in central China are used as adsorbents for the uptake of phosphorus from aqueous solutions. The X-ray fluorescence (XRF) spectrometric and BET (Brunauer-Emmett-Teller) tests and the Scanning Electron Microscopy (SEM) and Fourier Transform Infrared (FTIR) spectral analyses are carried out to investigate the soils’ chemical properties and their potential changes with adsorbed phosphorous from aqueous solutions. The intra-particle diffusion, pseudo-first-order, and pseudo-second-order kinetic models describe the adsorption kinetic processes. Our results indicate that the adsorption processes of phosphorus in soils occurred in three stages and that the rate-controlling steps are not solely dependent on intra-particle diffusion. A quantitative comparison of two kinetics models based on their linear and non-linear representations, and using the chi-square (*χ*^2^) test and the coefficient of determination (*r*^2^), indicates that the adsorptive properties of the soils are best described by the non-linear pseudo-second-order kinetic model. The adsorption characteristics of aqueous phosphorous are determined along with the essential kinetic parameters.

## 1. Introduction

The safety of drinking water supplies has been seriously threatened by water eutrophication. The primary cause of eutrophication is the oversupply of nutrients, which induces explosive growth of certain types of harmful algae and, consequently, disturbs aquatic ecosystems [1,2,3,4,5]. The excessive input of phosphorus is the most common cause of eutrophication of fresh water reservoirs [6,7].

The three-route (East, Middle and West) South-to-North Water Transfer Project (SNWTP) was implemented in 2002 to mitigate the increasing water resource deficits in north China [8,9]. The Middle Route Project (MRP) would transfer about 14 billion m^3^ of water annually from the Danjiangkou reservoir on the Han River, which is a large tributary to the middle reaches of the Yangtze River, to Beijing and Tianjin. More and more studies have been carried out to preserve public health in the Han River basin and develop a water conservation strategy for SNWTP [10,11,12,13]. Han River and Danjiangkou reservoir’s water quality aquatic ecosystem health is significant for the fate of the MRP water transfer project. Phosphorus is one of the primary pollutants in the rivers along the water conveyance system of MRP [14]. Algal blooms and increasing phosphorus concentrations have been observed in several tributary estuaries and bays of the Danjiangkou Reservoir in recent years, and these phenomena are unfavorable for the MRP [9,15,16]. Therefore, a series of ecological treatment and restoration approaches, such as ecological filtration dams, riparian zones/areas or constructed wetlands, are proposed to be constructed to reduce nutrients from agricultural runoff and to control eutrophication for the Danjiangkou Reservoir area [17]. In these treatment systems, influents are temporarily contained and treated within vegetated areas using (wherever possible) on-site soils. The main form of phosphorus in natural water bodies is phosphate. Therefore, the adsorption of phosphates plays an important role in phosphorus removal and retention in those ecological treatment and restoration systems.

For the purpose of optimally designing and controlling treatment processes of aqueous phosphorous, it is important to model its adsorption rate, which determines the residence time required for completion of the pertinent reactions, and to simulate the kinetic and dynamic behavior of pollutants on the adsorbents [18]. Several mathematical models have been developed to describe the adsorption kinetics in batch systems. Those models can be generally classified as adsorption reaction models and a diffusion model [19,20,21,22,23]. The first-order rate equation, presented by Lagergren at the end of 19th century [20], is the most popular empirical model used to describe the kinetic process of liquid-to-solid adsorption. In 1995, a pseudo-second-order kinetic expression was described by Ho, and this model has been used in many adsorption systems [22]. In addition, adsorption-diffusion models have been developed to describe the diffusion mechanism and rate controlling steps that affect the adsorption of aqueous pollutant [23].

This work investigates the time-dependent adsorption characteristics of aqueous phosphorus in eight soil types having wide-ranging physicochemical properties and found in diverse representative ecological zones. The soils have potential use as ecological restoration sites in a source area for drinking water in central China, that is, the Danjiangkou Reservoir. Diffusion and kinetic models as well as adsorption rate constants are used to describe the adsorption characteristics of aqueous phosphorus. This work’s findings have significance for assessing the feasibility of using on-site natural soils in the ecological treatment and restoration pilot applications of phosphorous removal.

## 2. Materials and Methods

### 2.1. Sampling Site Description

Eight soil types were obtained from surface soils (0–20 cm deep) from different representative ecological zones in the Danjiangkou reservoir area in central China. Three to five replicate soil samples were retrieved randomly at each site. This region has a subtropical monsoonal climate. The average annual precipitation is approximately 900 mm and 70%–80% of the precipitation occurs between April and October, the mean annual air temperature ranges from 12 °C–16 °C. Table S1 shows the sampling locations for eight soils: orchard soils (OS), forest soils (FS), natural grassland soils (NGS), abandoned cropland soils (ACS), furrowed cropland soils (FCS), cultivated cropland soils (CCS), riparian soils (RS), and bare soils (BS), respectively.

### 2.2. Pretreatment and Analysis

Soil samples were air-dried, prescreened to remove large gravel particles and woody fragments, and crushed gently and sieved to particle size <0.15 mm prior to the adsorption experiments. The samples chemical composition analysis was carried out using an X-ray fluorescence (XRF) spectrometer Bruker AXS S4 Pioneer. A N_2_ gas BET (Brunauer-Emmett-Teller) analysis was made by using a Micromeritics Chemisorption ASAP 2020. The Scanning Electron Microscopy (SEM) measurement was carried out using a FEI Quanta 200 apparatus. Fourier Transform Infrared (FTIR) spectra were recorded in the 400 cm^−1^–4000 cm^−1^ range, and measured with a 4 cm^−1^ resolution, using a Nicolet 5700 FTIR spectrometer.

### 2.3. Adsorption Experimental Procedure

A beaker (5 L) was prepared by mixing 40 g of dried soil adsorbents, which were oven-dried at 170 °C for 2 h before experiments to inhibit the microbial activity, and 1L liquid solution with initial phosphorus concentrations equal to 10 mg/L (0.02 mol/L KCl was added as electrolyte to each solution). The reaction occurred at a constant temperature of 25 °C and at a constant agitation speed of 170 rpm. Five milliliters of slurry was pipetted out at different times and centrifuged immediately for 1 min at 10,000 rpm at 25 °C in a HITACHI CR22G II High-speed Refrigerated Centrifuge and then filtered to completely separate the liquid and solid phases. The remaining phosphorus concentration was measured according to the ammonium molybdate spectrophotometric method with a spectrophotometer.

### 2.4. Adsorption Kinetic Model

The kinetics of adsorption of phosphorus onto soil adsorbents were analyzed using the intra-particle diffusion, pseudo-first-order model and pseudo-second-order models. Statistical criteria used for estimating the goodness-of-fit of the models to the experimental data were the coefficients of determination (*r*^2^).

The intra-particle diffusion model, developed to describe the diffusion mechanism and rate controlling steps that affect the adsorption process, is expressed by Equation (1) [23].

(1) $$q_{t}=k_{P}t^{1/2}+C$$

where *k_P_* is the inter particle diffusion rate constant (mg/(g·min^1/2^)), and *C* is the intercept (mg/g).

The pseudo-first-order kinetic model, presented by Lagergren, is expressed by Equation (2) [20]:

(2) $$\frac{\text{d}q_{t}}{\text{d}t}=k_{1}(q_{e}-q_{t})$$

Integrating Equation (2) between the limits from initial conditions *q_t_* = 0 at *t* = 0 to *q_t_* = *q_t_* at time *t* yields:

(3) $$q_{t}=q_{e}\left[1-\text{exp}(-k_{1}t)\right]$$

where *k*_1_ is the rate constant of adsorption (g/min), *q_e_* is the amount of phosphorus adsorbed at equilibrium (mg/g), and *q_t_* is the amount of phosphorus adsorbed at time *t* (mg/g).

Ho’s pseudo-second-order kinetic model is expressed by Equation (4) [21,22]:

(4) $$\frac{\text{d}q_{t}}{\text{d}t}=k_{2}{\left(q_{e}-q_{t}\right)}^{2}$$

For initial conditions *q_t_* = 0 at *t* = 0 the integrated form of Equation (4) is:

(5) $$q_{t}=\frac{q_{e}^{2}k_{2}t}{1+q_{e}k_{2}t}$$

where *k*_2_ is the rate constant of adsorption (g/(mg·min)), *q_e_* is the amount of phosphorus adsorbed at equilibrium (mg/g), and *q_t_* is the amount of phosphorus adsorbed at time *t* (mg/g).

## 3. Results and Discussion

### 3.1. Elemental Composition Analysis

The chemical compositions of eight soils from diverse ecological zones in the Danjiangkou reservoir area were characterized using XRF spectrometric analyses. Table S1 demonstrate similar elemental compositions for the eight types of soil, which are mainly composed of O (45.45%–50.57%), Si (23.83%–30.45%), Al (8.150%–10.56%), Fe (2.436%–9.776%) and Ca (0.748%–10.64%).

### 3.2. Surface Textural Property Analysis

To gain further insights into the porous nature of the studied soil adsorbents, BET measurements were performed to examine their surface textural properties. Although all eight soils were derived from diverse ecological zones, their adsorption-desorption isotherms for N_2_ are similar (Figure S1). Generally, all eight isotherms are of the Type IV isotherms with Type H4 hysteretic loops, which are often associated with narrow slit-like pores and exhibited many mesoporous adsorbents. The initial part of each curve, which follows the same path as the corresponding part of the Type II isotherm, is attributed to monolayer-multilayer adsorption; the point B, at the beginning of the almost linear middle section of the isotherm, indicates the stage at which monolayer coverage is complete and multilayer adsorption is about to begin. Its hysteresis loop corresponds to the Type H4 loop because of the unlimited adsorption at high relative pressure p/p_0_ (equilibrated pressure/saturation pressure) [24]. Moreover, it can be seen that the adsorption–desorption hysteresis loops for all eight curves closed at a similar relative pressure (p/p_0_: 0.45). This kind of feature can be also found for other soils [25]. The pore diameters, BET surface areas and total pore volumes obtained from the adsorption–desorption isotherms of N_2_ are listed in Table S2. Most pores in the studied soil samples were sized at about 4.90–10.13 nm in diameter. The BET surface areas varied from 37.88 m^2^/g–1.93 m^2^/g, with decreasing values in the order OS > CCS > NGS > FCS > ACS > RS > BS > FS. The total pore volumes of the eight soils ranged from 0.0509 cm^3^/g–0.0070 cm^3^/g in increasing order for the eight soils in the sequence CCS > FCS > NGS > OS > ACS > RS > BS > FS. Soil OS had the largest BET surface area and soil CCS had the largest total pore volume, while soil FS had the smallest BET surface area and total pore volume.

### 3.3. SEM and FTIR Analysis

**Figure 1:**
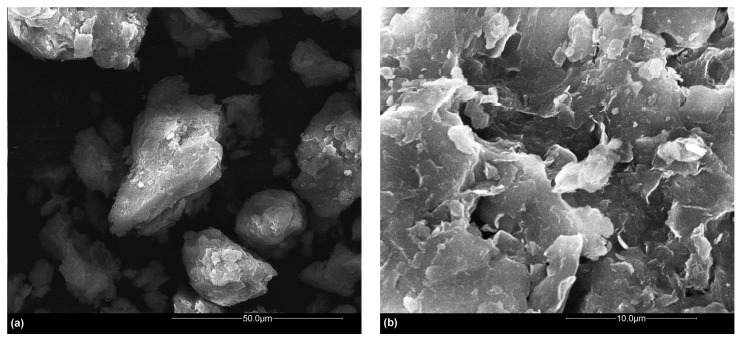

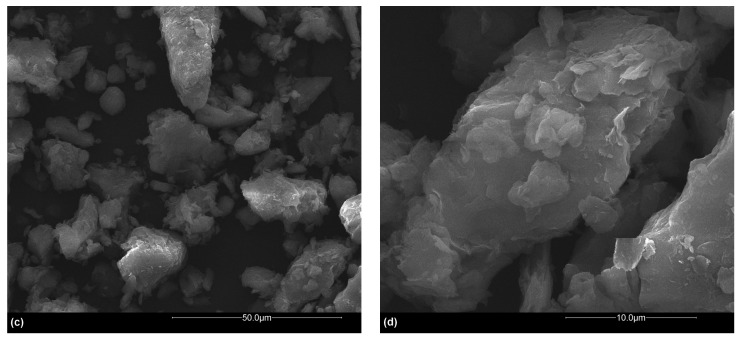
Scanning electronic microscope (SEM) images of (**a**) original cultivated cropland soil sample at working distances of 50 µm; and (**b**) 10 µm; and (**c**) phosphorus solution treated soil sample at working distances of 50 µm; and (**d**) 10 µm.

The surface morphology and chemical make-up features of soil samples and their potential changes with the adsorption of phosphorus were also investigated. Before and after the adsorbent phosphorus was extracted from aqueous solution, soil CCS (which has a relatively large BET surface area and total pore volume) was selected as an example of the soil samples and examined by SEM and FTIR analyses. It was found that the original soil sample had a rough surface with irregularly distributed gaps (Figure 1a,b). The addition of phosphorus reduced the spacing between minerals slightly but did not significantly change the surface morphology (Figure 1c,d). It might be because the gaps were occupied by the generated small complex compounds with the adsorption of phosphorus [26].

The FTIR spectrum of the CCS soil sample (Figure S2) shows obvious water bending modes at 1639 m^−1^ accompanied by OH stretching vibrations at 3425–3621 cm^−1^. A weak band near 1432 cm^−1^ is characteristic of calcium carbonate. The broad band around 1031 cm^−1^ can be assigned to Si-O stretching vibrations. The double-peak bands near 779 cm^−1^ are assigned to the symmetric stretching vibration of O-Si-O in soil. The Si-O-Moct (Moct = Fe, Al, and Mg) bending vibrations bands appear within the 400–600 cm^−1^ region [27]. The phosphorus adsorbed FTIR spectrum of the CCS soil sample is shown in Figure S2b. The slight shifts at peaks 1432 and 400–600 cm^−1^ indicate that those microstructures may be involved in the extracting of phosphorus during the adsorption process.

### 3.4. Effect of Agitation Time

The effect of contact time on the amount of phosphorus adsorbed was investigated in a batch reactor with rapid stirring. The extent of removal of phosphorus by all eight soil adsorbents was found to increase and then gradually approach a more or less constant value with the increase in agitation time. Similarly, a rapidly increasing adsorption in the first 10 min for each soil adsorbent was observed, and the uptake of phosphorus after 20–40 min approached a constant asymptotic value, denoting attainment of equilibrium. However, the initial adsorption rates of eight soil adsorbents varied. Therefore, the intra-particle diffusion, pseudo-first-order, and pseudo-second-order models were introduced in this study to investigate their adsorption kinetics and test the adsorption mechanism subsequently.

### 3.5. Intra-Particle Diffusion Study

The adsorption process for porous solid adsorbents can be described by four steps: (1) transport in the bulk of the solution; (2) external binding or boundary layer diffusion; (3) intra-particle diffusion; and (4) adsorption and desorption within the particle and on the external surface [18,28,29,30]. One or more of those four steps may be involved in the rate-controlling step. In the present adsorption system, the transport in the solution could be ignored since the adsorption process occurred in a rapidly stirred batch reactor, which eliminated the effect of the movement of the solute from the bulk liquid film surrounding the adsorbent. Moreover, it was found that the intra-particle diffusion is often the rate-controlling step in many adsorption processes in a batch reactor with rapid stirring [18,31].

**Figure 2:**
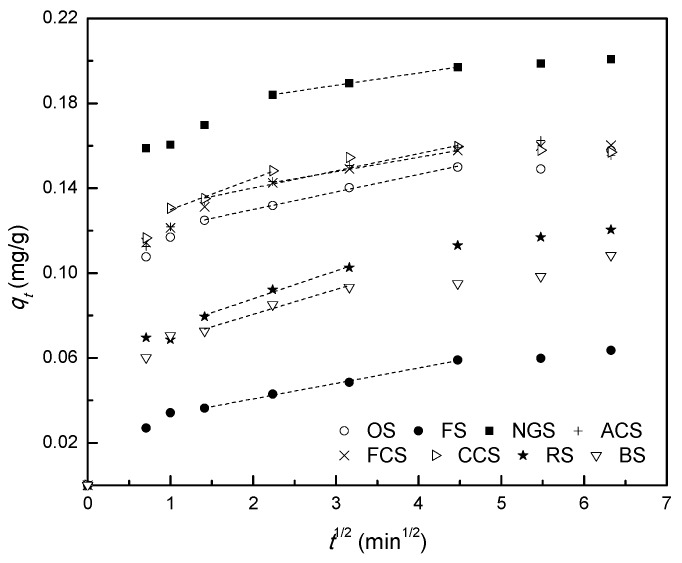
Intra-particle diffusion adsorption kinetics of phosphorus on soil. Notes: OS: orchard soils; FS: forest soils; NGS: natural grassland soils; ACS: abandoned cropland soils; FCS: furrowed cropland soils; CCS: cultivated cropland soils; RS: riparian soils; BS: bare soils.

The diffusion mechanism and rate-controlling steps that affected the adsorption process were explored with the intra-particle diffusion model [23]. The amount of phosphorus adsorbed (*q_t_*) onto different soil adsorbents against the square root of the time of uptake (*t*^1/2^) was investigated. If the intra-particle diffusion was involved in the adsorption process, the plot of *q_t_ vs. t*^1/2^ would yield a straight line, and, if the rate-limiting step was only intra-particle diffusion, this line would pass through the origin [18,32]. The plots of *q_t_ vs. t*^1/2^ for eight soil adsorbents are shown in Figure 2, it demonstrates that all the plots feature three separate stages: (1) an initial curved portion; (2) an intermediate linear portion; and (3) a plateau starts after 20 min. The initial portion is attributed to the boundary layer effects (external mass transfer), the second linear portion due to intra-particle diffusion, and the plateau portion to the final equilibrium stage where intra-particle diffusion starts to slow down [33]. These three stages have also been observed in another adsorption process of phosphorus, in which calcined alunite was used as adsorbent [34].

The values of the rate constant for intra-particle diffusion, *k_id_*, are obtained from the slopes of the lines in stage 2 (Table 1). The value of *k_id_* is minimum for soil NGS (0.0058 mg/g·min^1/2^) and maximum for soil CCS (0.0146 mg/g·min^1/2^) with the experimental adsorption setup. As shown in Table 1, the intercepts, *C*, which are associated positively with the boundary layer effect, are also calculated from the second, linear portions of the plots of *q_t_ vs. t*^1/2^ [31,35]. Generally, the deviations of the linear portions of the plots of *q_t_ vs. t*^1/2^ by the eight soil adsorbents near the origin are obvious. It may be concluded that the adsorption mechanism of phosphorus on soils from diverse ecological zones in Danjiangkou reservoir area is complex and that the adsorption process is not solely rate-controlled by the intra-particle diffusion step.

**Table 1:** Intra-particle diffusion rate constants and intercept values for the adsorption of phosphorus onto soil.

Soil	*k_id_* (mg/g·min ^1/2^)	*C* (mg/g)	*r*^2^
OS	0.0082	0.1134	0.9979
FS	0.0073	0.0261	0.9967
NGS	0.0058	0.1710	1.0000
ACS	0.0081	0.1239	0.9831
FCS	0.0068	0.1275	0.9997
CCS	0.0146	0.1153	0.9936
RS	0.0131	0.0616	0.9921
BS	0.0117	0.0570	0.9764

Notes: OS: orchard soils; FS: forest soils; NGS: natural grassland soils; ACS: abandoned cropland soils; FCS: furrowed cropland soils; CCS: cultivated cropland soils; RS: riparian soils; BS: bare soils.

### 3.6. Pseudo-First-Order and Pseudo-Second-Order Kinetic Study

The pseudo-first-order and pseudo-second-order models are commonly compared and applied in their linearized forms, and the least squares method is always used for determining the best-fitting models and obtaining the kinetic parameters. [21]. The pseudo-first-order model (Equations (2) and (3)) can be rearranged for linearized data plotting as follows (Equation (6)):

(6) $$\text{log}\left(q_{e}-q_{t}\right)=\text{log}\left(q_{t}\right)-\frac{k_{1}}{2.303}t$$

The plots of log(*q_e_* − *q_t_*) versus *t* for the adsorption of phosphorus onto eight soils were tested. Linear fitness examination of the pseudo-first-order rate plots and the calculated *r*^2^ equal to 0.8050, 0.8760, 0.6445, 0.5970, 0.6852, 0.5978, 0.8187, or 0.7658 confirmed that the adsorption kinetics did not fit well with this model for soils OS, FS, NGS, ACS, FCS, CCS, RS, or BS, respectively. The pseudo-second-order model (Equations (4) and (5)) can be also linearized as four different types, and the constants of the model are calculated experimentally by plotting *t*/*q_t_ versus t*, 1/*q_t_ versus* 1/*t*, *q_t_ versus q_t_*/*t*, and *q_t_*/*t versus q_t_* for linear equation type 1 (Equation (7)), type 2 (Equation (8)), type 3 (Equation (9)), and type 4 (Equation (10)), respectively [22].

(7) $$\frac{t}{q_{t}}=\frac{1}{k_{2}q_{e}^{2}}+\frac{t}{q_{e}}$$

(8) $$\frac{1}{q_{t}}=\left(\frac{1}{k_{2}q_{e}^{2}}\right)\frac{1}{t}+\frac{1}{q_{e}}$$

(9) $$q_{t}=q_{e}-\left(\frac{1}{k_{2}q_{e}}\right)\frac{q_{t}}{t}$$

(10) $$\frac{q_{t}}{t}=k_{2}q_{e}^{2}-k_{2}q_{e}q_{t}$$

**Figure 3:**
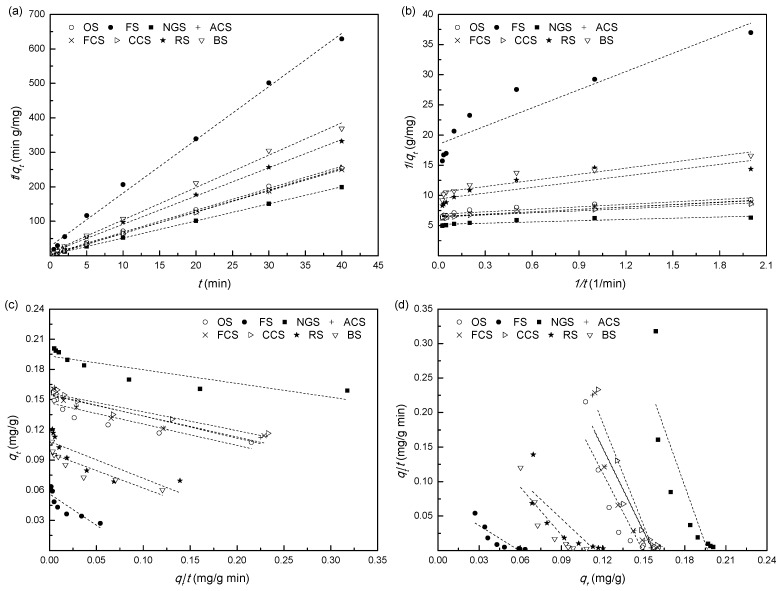
Pseudo-second-order kinetic plots for four types of linear equations (**a**–**d**) for the adsorption of phosphorus on soils. Notes: OS: orchard soils; FS: forest soils; NGS: natural grassland soils; ACS: abandoned cropland soils; FCS: furrowed cropland soils; CCS: cultivated cropland soils; RS: riparian soils; BS: bare soils.

Figure 3 depicts the variation of the pseudo-second-order kinetic plots using the linear least squares method for the adsorption of phosphorus on the eight experimental soils. The values of the calculated constants *q*_e_, *k*_2_, and the *r*^2^ for four expression types were significantly different. Linear fitness examination of the pseudo-second-order rate plots revealed that the type 1 linear equation yielded the best results with high *r*^2^ values (0.9951–0.9999) (Figure 3a). The calculated parameters of the type 1 linear equation are shown in Table 2. This type 1 linear equation is the most commonly used linear form of the pseudo-second-order model. It has been preferably used to describe the kinetic characteristics of many adsorption processes [21,36]. The non-linear regression method was also applied in this paper to determine the best-fitting equation and obtain the kinetic parameters, and to compare its results with those obtained with the linear method for pseudo-first-order and pseudo-second-order models. The chi-squared (*χ*^2^) test was used as the statistical criteria to quantitatively compare the applicability of each kinetic equation, and as the *r*^2^ for estimating the goodness-of-fit of the models to the experimental data [37,38]. The chi-squared statistic is calculated as follows:

(11) $$\chi^{2}=\sum \frac{{\left(q_{t\text{,}\exp}-q_{t\text{,cal}}\right)}^{2}}{q_{t\text{,cal}}}$$

Table 2 lists the calculated results of the pseudo-first-order and pseudo-second-order models obtained with non-linear regression using the software SOLVER available in EXCEL. As an example, Figure S3 portrays a fitting result obtained with the implemented experimental adsorption set up (example only, soil CCS). In Table 2, the high *r*^2^ and low *χ*^2^ values, for all eight soil adsorbents obtained using the non-linear method, indicate that the pseudo-second order kinetic model gives an agreement between the experimental and calculated adsorption capacity values that is better than that obtained with the linear model. The comparison results between the pseudo-first-order model and the pseudo-second order model obtained with linear and non-linear methods is consistent: the pseudo-second order model is more suitable for the description of the kinetic nature of phosphorus adsorption on each soil adsorbent. The experimental data of soil CCS was fitted best with the pseudo-second order model, with highest *r*^2^ and lowest *χ*^2^ of 0.9948 and 0.0008 by the non-linear method, and 0.9999 and 0.0011 by the linear method.

**Table 2:** Comparison of parameters obtained with different kinetic equations.

Soil	*q*_e,exp_	Pseudo-First-Order (Non-Linear)				Pseudo-Second-Order (Non-Linear)				Pseudo-Second-Order (Linear Type 1)			
		*k*_1_	*q*_e,cal_	*r*^2^	*χ*^2^	*k*_2_	*q*_e,cal_	*r*^2^	*χ*^2^	*k*_2_	*q*_e,cal_	*r*^2^	*χ*^2^
OS	0.162	2.28	0.143	0.9499	0.0072	28.2	0.148	0.9807	0.0027	9.28	0.157	0.9985	0.0335
FS	0.073	0.68	0.057	0.8888	0.0140	16.8	0.060	0.9446	0.0054	8.72	0.065	0.9957	0.0180
NGS	0.212	3.13	0.189	0.9635	0.0065	33.2	0.195	0.9873	0.0023	13.0	0.202	0.9999	0.0186
ACS	0.170	2.20	0.151	0.9619	0.0059	26.6	0.157	0.9896	0.0016	20.0	0.159	0.9990	0.0029
FCS	0.172	2.27	0.151	0.9549	0.0069	27.2	0.156	0.9862	0.0021	12.6	0.162	0.9998	0.0149
CCS	0.162	2.54	0.152	0.9743	0.0037	32.3	0.157	0.9948	0.0008	27.5	0.159	0.9999	0.0011
RS	0.135	1.05	0.110	0.8960	0.0213	15.2	0.115	0.9508	0.0083	7.03	0.122	0.9986	0.0330
BS	0.120	1.41	0.095	0.9229	0.0092	23.3	0.099	0.9677	0.0035	8.64	0.107	0.9951	0.0297

Notes: OS: orchard soils; FS: forest soils; NGS: natural grassland soils; ACS: abandoned cropland soils; FCS: furrowed cropland soils; CCS: cultivated cropland soils; RS: riparian soils; BS: bare soils.

As shown in Table 2, the parameters of the pseudo-second-order model obtained with linear or non-linear methods vary widely. The adsorption rate constants for eight soils ranged from 7.03–27.5 g/(mg·min) when using the linear regression method, and the phosphorus adsorption rates were found to decrease in the following order for the eight experimental soils: RS < BS < FS < OS < FCS < NGS < ACS < CCS. The adsorption rate constants vary between 15.2 and 33.2 g/(mg·min) while the adsorption rates decreased in the following order RS < FS < BS < ACS < FCS < OS < CCS < NGS when the non-linear method was used. Moreover, for each soil adsorbent, the *r*^2^ and the *χ*^2^ values obtained with the linear method were larger than those obtained with the non-linear method (Table 2). This anomaly (that is, *r*^2^ and *χ*^2^ being simultaneously larger with the linear method than with nonlinear method) might be caused by the transformation of the non-linear pseudo-second-order kinetic model to its linear form. This kind of transformation might implicitly alter the model’s error structure and violate the error variance and normality assumptions of the standard least squares method [22,39]. The determination process of *r*^2^ was significantly influenced, but the *χ*^2^ test avoided this error [40]. The modeling-predicted data with the nonlinear method approximated the experimentally measured data, yielding low *χ*^2^ values, even though the values of phosphorus adsorbed at equilibrium (*q*_e,cal_) calculated with the linear method were closer to the experimental (*q*_e,exp_) values (Table 2).

**Figure 4:**
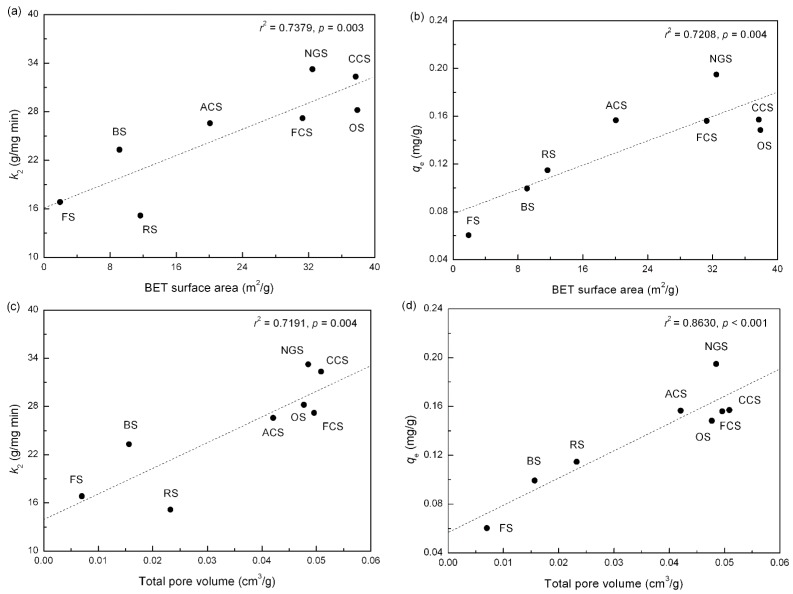
Variation of the non-linear pseudo-second-order kinetic constants as a function of surface textural characteristics of soils (**a**–**d**). Notes: OS: orchard soils; FS: forest soils; NGS: natural grassland soils; ACS: abandoned cropland soils; FCS: furrowed cropland soils; CCS: cultivated cropland soils; RS: riparian soils; BS: bare soils.

The adsorption rate and capacity may be affected by the physical and chemical properties of the adsorbent, such as the porosity (a physical property). The non-linear pseudo-second-order kinetic constants, *k*_2_ and *q*_e_, are plotted against the main surface textural characteristics of the soil adsorbents in Figure 4. Linear positive correlations, with BET surface area *versus k*_2_ (*r*^2^ = 0.7379, *p* = 0.003), BET surface area *versus q*_e_ (*r*^2^ = 0.7208, *p* = 0.004), and total pore volume *versus k*_2_ (*r*^2^ = 0.7191, *p* = 0.004), total pore volume *versus q*_e_ (*r*^2^ = 0.8630, *p* < 0.001), indicate that the adsorption rates and capacities of phosphorus are directly proportional to the porosity of the studied soil adsorbents. Therefore, high adsorption rates and capacities could be obtained on those fine-textured soils offering active adsorption sites in the Danjiangkou reservoir area.

## 4. Conclusions

The time-dependent adsorption behavior of phosphorus for eight soils, collected from diverse representative ecological zones in the source area of drinking water of a reservoir in central China, was investigated in this paper. A similar elemental composition and surface textural nature was found for all eight soils. No significant change in the surface morphology and chemical make-up features of the studied soil with the addition of phosphorus was observed.

The adsorption processes of phosphorus for all eight soils exhibited three stages, instead of a rate-controlled process by intra-particle diffusion. A contradiction between diagnostic criteria (*r^2^* and *χ*^2^) was caused by the transformation of the non-linear kinetic model to its linear form. This anomaly could be avoided if the chi-square (*χ*^2^) test were used as the sole statistical criterion for comparing different linear and non-linear kinetic equations.

The non-linear pseudo-second-order kinetic model was more suitable for describing the adsorption behavior of phosphorus in soils from various ecological zones in the Danjiangkou reservoir area. The calculated adsorption rates of eight soils ranged from 15.2–33.2 g/(mg·min) with decreasing order RS < FS < BS < ACS < FCS < OS < CCS < NGS, and the simulated adsorption capacities ranged from 0.060–0.195 mg/g with decreasing order FS < BS < RS < OS < FCS < ACS < CCS < NGS. Moreover, it was found that the adsorption rate and capacity of phosphorus were directly related to the porosity of the soil adsorbents. This result suggests that fine-textured soils have definite potential as substrate materials used in the ecological treatment and restoration systems for the removal and retention of phosphorus.

## Supplementary Files

Supplementary File 1
