# Supplementary material for: Kinetics and Mechanisms of Phosphorus Adsorption in Soils from Diverse Ecological Zones in the Source Area of a Drinking-Water Reservoir
Source: Int J Environ Res Public Health. 2015 Nov 10;12(11):14312–26. doi: 10.3390/ijerph121114312 (PMC4661649; doi:10.3390/ijerph121114312)
Supplement: Supplementary File 1 [file ijerph-12-14312-s001.pdf]

Supplementary Information

Kinetics and Mechanisms of Phosphorus Adsorption on Soils from Diverse Ecological Zones in the Source Area of a Drinking-Water Reservoir

Table S1. Sampling locations and main chemical composition of soil samples.

Soil	Sampling Location			Main Element Composition (wt. %)							
	Longitude	Latitude	Description	O	Si	Al	Fe	Ca	Na	K	Mg
OS	111°08.253′	32°42.609′	orchard land	48.58	30.45	10.25	4.951	0.748	0.507	2.464	1.076
FS	111°25.486′	32°34.247′	forest land	50.57	29.31	8.872	2.436	0.848	1.730	2.478	0.894
NGS	111°08.142′	32°42.823′	natural grassland	48.24	28.64	10.22	4.967	1.723	0.574	2.782	1.365
ACS	111°09.570′	32°42.658′	abandoned cropland	47.75	27.62	10.56	4.999	2.288	0.636	3.192	1.542
FCS	111°27.329′	32°41.606′	furrowed cropland	46.66	24.15	8.150	3.931	10.64	0.281	2.126	1.978
CCS	111°27.372′	32°41.693′	cultivated cropland	48.77	29.35	9.288	4.405	1.798	0.420	2.604	1.726
RS	111°27.794′	32°34.948′	riparian wetland	46.78	27.12	9.513	7.036	3.878	1.960	1.050	1.927
BS	111°27.797′	32°34.970′	bare land	45.45	23.83	9.917	9.776	4.237	1.880	0.476	3.718

Notes: OS: orchard soils; FS: forest soils; NGS: natural grassland soils; ACS: abandoned cropland soils; FCS: furrowed cropland soils; CCS: cultivated cropland soils; RS: riparian soils; BS: bare soils.

Table S2. Surface textural properties of soil samples.

Soil	Average Pore Diameter (nm)	BET Surface Area (m^2/g)	Total Pore Volume (cm^3/g)
OS	4.90	37.88	0.0477
FS	10.13	1.93	0.0070
NGS	5.30	32.47	0.0485
ACS	7.39	20.07	0.0421
FCS	6.35	31.27	0.0496
CCS	5.32	37.70	0.0509
RS	6.12	11.66	0.0232
BS	5.63	9.12	0.0156

Notes: OS: orchard soils; FS: forest soils; NGS: natural grassland soils; ACS: abandoned cropland soils; FCS: furrowed cropland soils; CCS: cultivated cropland soils; RS: riparian soils; BS: bare soils.

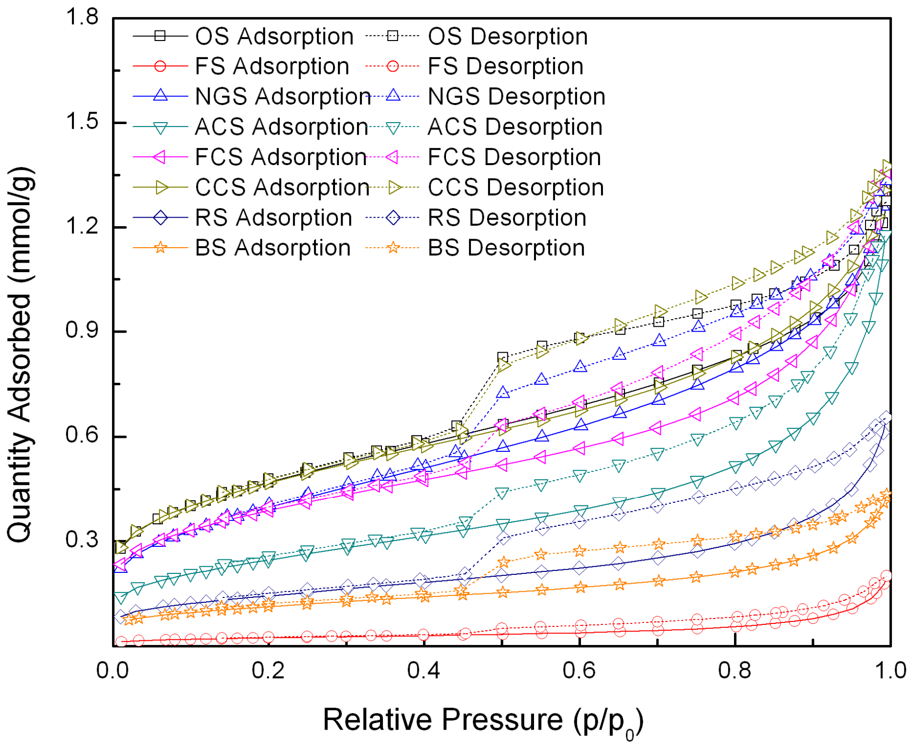

Figure S1. Adsorption-desorption isotherms of N_2 on soil samples. Notes: OS: orchard soils; FS: forest soils; NGS: natural grassland soils; ACS: abandoned cropland soils; FCS: furrowed cropland soils; CCS: cultivated cropland soils; RS: riparian soils; BS: bare soils.

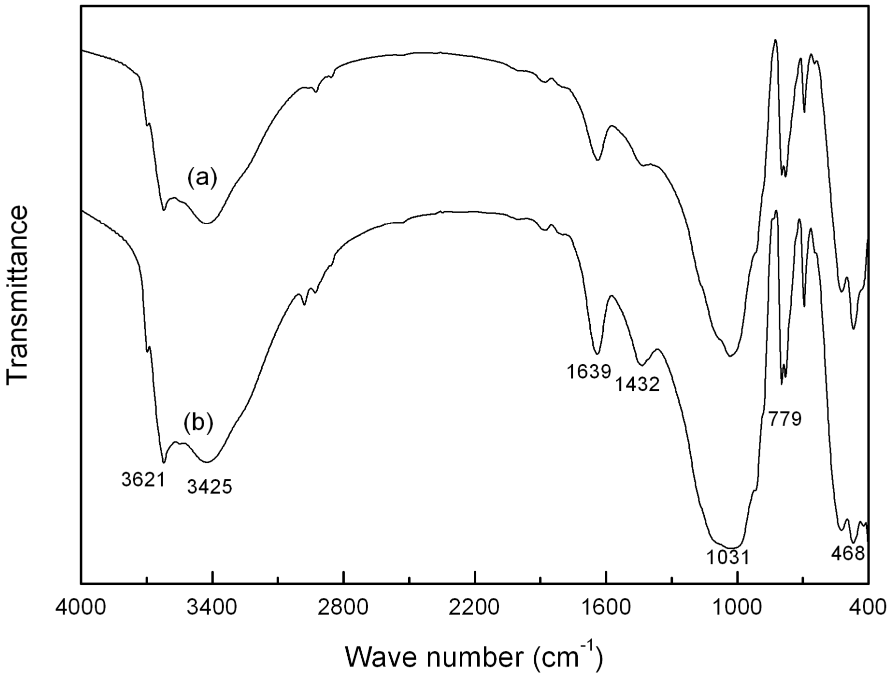

Figure S2. Fourier Transform infrared (FTIR) spectra of (**a**) original CCS soil sample; and (**b**) phosphorus solution treated soil sample. Notes: CCS: cultivated cropland soils.

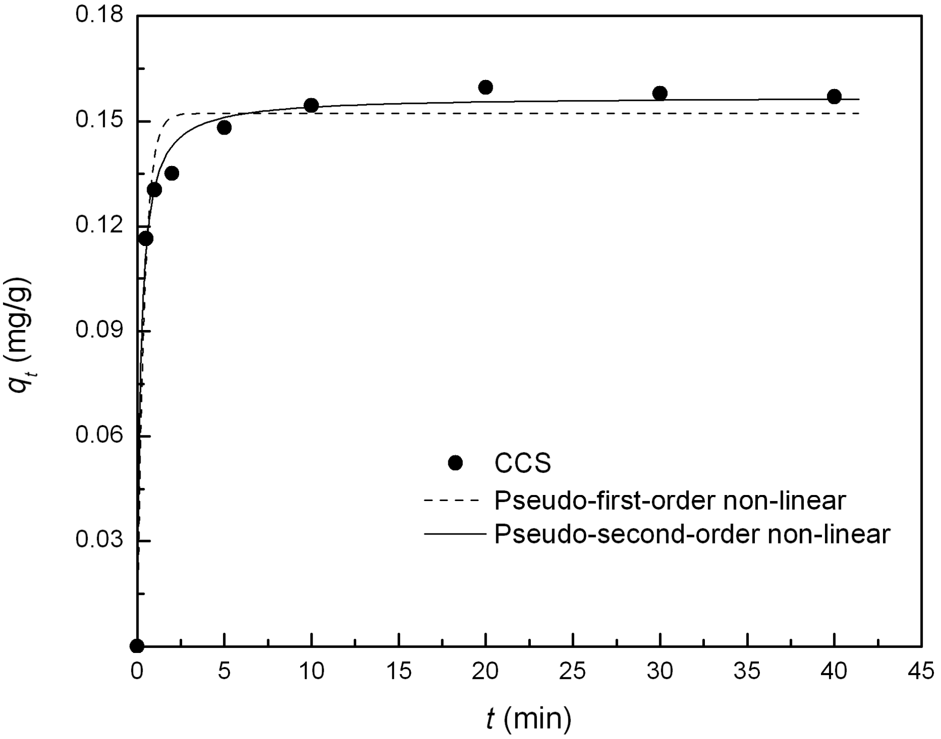

Figure S3. Non-linear pseudo-first-order and pseudo-second-order kinetic plot of phosphorus adsorption on soil CCS. Notes: CCS: cultivated cropland soils.

© 2015 by the authors; licensee MDPI, Basel, Switzerland. This article is an open access article distributed under the terms and conditions of the Creative Commons Attribution license (http://creativecommons.org/licenses/by/4.0/).
